# Targeting interleukin-1β and inflammation in lung cancer

**DOI:** 10.1186/s40364-021-00341-5

**Published:** 2022-01-27

**Authors:** Jun Zhang, Nirmal Veeramachaneni

**Affiliations:** 1 Division of Medical Oncology, Department of Internal Medicine, Department of Cancer Biology, University of Kansas Medical Center, 3901 Rainbow Blvd, Kansas City, KS 66160 USA; 2 Department of Cardiovascular and Thoracic Surgery, University of Kansas Medical Center, 3901 Rainbow Blvd, Kansas City, KS 66160 USA

**Keywords:** Interleukin-1 beta, Lung cancer, Inflammation, Tumor microenvironment, Immunotherapy

## Abstract

Inflammation is a process that protects organs against various potentially harmful stimuli and enables repair. Dysregulated inflammation, however, damages tissues and leads to disease, including cancer. Cancer-related inflammation is characterized by cytokine production, leukocyte infiltration, angiogenesis, and tissue remodeling—all critical processes in modulating the tumor microenvironment (TME). The TME is known to play a key role in tumor progression, and targeting its immune component to achieve a better anti-tumor response is the basis of immunotherapy. Despite the critical role cytokines play in the TME and tumor progression, there is currently only one therapy approved by the FDA that directly involves cytokine signaling: human recombinant interleukin-2 protein, aldesleukin. The recent Canakinumab Anti-inflammatory Thrombosis Outcomes Study (CANTOS) trial evaluated the use of anti-interleukin-1β therapy in atherosclerotic disease; however, it also revealed interleukin-1β (IL-1β) blockade with canakinumab led to a significantly lower incidence of lung cancer. This has opened a promising new avenue for lung cancer therapy, and strategies using anti-IL-1β therapy alone or in combination with chemotherapy and/or immune checkpoint blockade are currently being evaluated in several clinical trials.

## Inflammation and cancer

### Inflammation in cancer initiation and progression

Inflammation is one of the hallmarks of cancer and plays a key role in mediating cancer initiation, cell proliferation, invasion, angiogenesis, and metastasis [1]. Inflammation is the response to stimuli of a heterogeneous nature, either physical (e.g., burn, trauma), chemical (alcohol, toxins), biological (cell damage), or infectious (bacteria, virus) [2]. These stimuli lead to activation and recruitment of inflammatory cells via production of cytokines (e.g., IL-6) and inflammatory proteins (e.g., C-reactive protein [CRP]) [2]. Acute inflammation is an injury-repairing process that is beneficial to organs (“good” inflammation); however, if not eventually resolved, it leads to chronic inflammation, tissue remodeling, and T-cell dysfunction (“bad” inflammation) [2, 3]. Chronic inflammation causes a number of diseases, such as chronic pancreatitis, chronic obstructive pulmonary disease (COPD), inflammatory bowel disease, and cancer [2, 4].

Cancer-related inflammation or pro-tumor inflammation is characterized by the presence of cytokines and chemokines, leukocyte infiltration, angiogenesis, and tissue remodeling. Inflammation can be intrinsic—elicited by the tumor—or extrinsic [5]. Certain stimuli can trigger chronic inflammation and induce carcinogenesis. For example, development of lung cancer is associated with COPD caused by smoking-related lung damage, inflammation, and subsequent DNA damage [4]. Liver cancer is associated with fibrosis and cirrhosis caused by the chronic inflammation resulting from alcohol or hepatitis virus B or C infection [4]. These represent extrinsic pathways that lead to inflammation [5]. Tumor-elicited inflammation is caused by the activation of oncogenes, which lead to cytokine production and tissue remodeling, and by the inactivation of tumor suppressor genes (e.g., PTEN, VHL) that also regulate production of inflammatory factors and cytokines [5]. Multiple preclinical models have implicated these stimuli in inducing genetic and epigenetic events that are required for cancer initiation and progression [6].

### Inflammation and tumor microenvironment

**Fig. 1:**
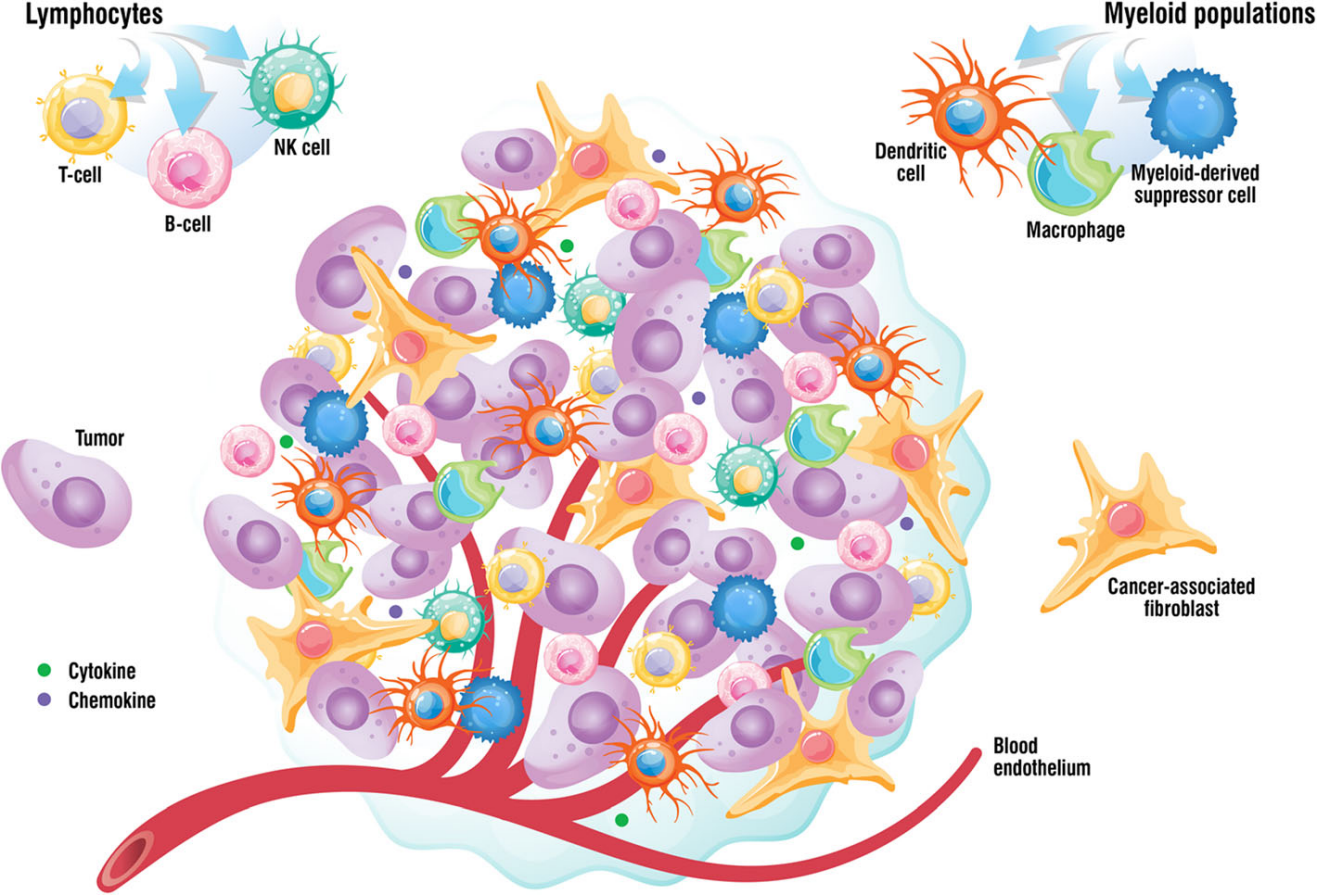
Main components of the tumor microenvironment. The tumor microenvironment comprises cellular and non-cellular fractions. The cellular component consists of cancer cells, endothelial cells, pericytes, carcinoma-associated fibroblasts, and immune cells. The immune compartment comprises several populations, such as B-cells, T-cells and natural killer cells, tumor-associated macrophages, myeloid-derived suppressor cells, and dendritic cells. The extracellular matrix represents the non-cellular component of the tumor microenvironment and acts as a scaffold. Elements of the tumor microenvironment interact via the extracellular matrix, cell-cell contacts, and through the release of cytokines, chemokines, and extracellular vesicles, among others. (Modified from Cui and Guo. 2016 [9])

Tumors are not solely composed of cancer cells. The TME comprises cellular and non-cellular components that play a critical role in tumor progression, response to therapy, and immune escape (Fig. 1) [1, 7, 8]. The extracellular matrix connects all components of the TME, acting like a scaffold, and is composed of fibrous proteins (e.g., collagen), proteoglycans, and laminins, among others [7].

Cells communicate dynamically through the extracellular matrix, and also via cell-cell contacts or through the release of cytokines, chemokines, enzymes, and extracellular vesicles such as exosomes, among others [7]. The main cell types found in the TME are cancer cells, cancer-associated fibroblasts (CAFs), pericytes, endothelial cells, and immune cells. CAFs originate from various cell types and promote the proliferation, migration, and survival of cancer cells, as well as induce angiogenesis and remodel the extracellular matrix. CAFs are also involved in creating an immunosuppressive environment [7, 10]. Pericytes remodel the basement membrane during angiogenesis and regulate lymphocyte activation [7]. Endothelial cells line blood vessels, regulate angiogenesis and immune cell infiltration, and also promote tumor progression and metastasis [7].

Immune cells in the TME play multiple roles that may change as the tumor progresses, as is the case of neutrophils that switch from an inflammatory to an immunosuppressive phenotype [8]. Opposing roles in cancer, either tumor-suppressive or tumor-promoting, have been found for immune cell types, such as CD4^+^ T-cells, CD8^+^ T-cells, natural killer (NK) cells, and macrophages [3]. Dendritic cells respond to cytokines in the environment, leading to adoption of a tolerogenic phenotype and secretion of inflammatory cytokines [8]. Myeloid-derived suppressor cells (MDSCs) also contribute to immunosuppression and increase the metastatic potential of cancer cells [8]. Tumor-associated macrophages are heterogeneous and play different roles in tumor invasion and angiogenesis based on their phenotype, which can be inflammatory (M1) or immunosuppressive (M2, tumor-promoting). The proportion of M2 macrophages is higher than that of M1 in the TME and is generally associated with poor prognosis [7, 8, 10].

Inflammation induced by various stimuli, including therapy, modulates the TME and can promote tumor growth and progression. Inflammation may also be responsible for response or resistance to therapy [11]. Cytokines in particular play a key role in the TME by mediating cancer progression [12]. IL-1, one of the main proinflammatory cytokines, is upregulated in many tumors and plays a role in inducing immunosuppression in the TME (more details in the following section) [13–15].

## Immunotherapy for cancer treatment

The immune component of the TME modulates the response to cancer treatment. Differences in the density, composition, functional state, and organization of immune cell subsets in the TME have been associated with prognosis [16]. Characterizing this immune contexture, together with other components (e.g., low infiltration by cytotoxic lymphocytes combined with high density of fibroblasts is linked to poor prognosis), may be useful in guiding the selection of appropriate immunotherapeutic strategies [17, 18]. Besides immune checkpoint blockade, immunotherapy strategies also include cytokine therapy, cellular therapy (e.g., CAR [chimeric antigen receptors] T-cell therapy), and therapeutic vaccines (e.g., autologous cellular immunotherapy for metastatic castrate- resistant prostate cancer) [12].

### Therapies targeting immune checkpoints

Immunotherapy has become a novel paradigm for cancer treatment fueled in part by the rapid understanding of the mechanisms that regulate immune surveillance and the discovery of immune checkpoints—immune-cell surface receptors that control the activation or inhibition of immune responses. Therapies based on immune checkpoint inhibition drive the activation of a better anti-tumor immune response. Checkpoint inhibitors against cytotoxic T-lymphocyte-associated protein 4 (CTLA-4), programmed death-1 (PD-1), or programmed death ligand 1 (PD-L1) are approved by the FDA for treating numerous types of cancer [12]. PD-1 is more broadly expressed than CTLA-4, and its expression is regarded as a hallmark of T-cell exhaustion [18, 19]. In tumor development, the PD-1/PD-L1 pathway leads to an inhibition of the host’s anti-tumor immunity, resulting in tumor immune escape. Based on the role this pathway plays in the TME, combination treatments with anti-PD-1/PD-L1 agents are being explored in a variety of cancers.

### Therapies targeting interleukins

Currently, the only FDA-approved drug for cancer based on the role of interleukins is aldesleukin, a human recombinant interleukin-2 product, used for metastatic melanoma and metastatic renal cell carcinoma [20]. Drugs targeting IL-1, namely canakinumab, anakinra, and rilonacept, are approved by the FDA for treating autoinflammatory diseases, such as periodic fever syndromes, and rheumatoid or systemic juvenile idiopathic arthritis [22–24]. Although clinical trials using anakinra for blocking IL-1 signaling in breast cancer [24], colorectal cancer [25], and indolent multiple myeloma [26, 27], in combination with other treatments, found it improved survival, decreased serum levels of CRP, downregulated components of the systemic inflammatory response, and increased cytotoxic activity, no anti-IL-1 therapy has been approved for cancer to date. However, ongoing clinical trials are studying anti-IL-1 strategies for treating cancer—pancreatic, breast, or renal, among others—either alone or in combination with chemotherapy or other immunotherapeutic agents, such as immune checkpoint inhibitors (Table 1). One such inhibitor is the anti-PD-1 agent spartalizumab (PDR001), which is still in the experimental phase of development [28, 29].

**Table 1:** Ongoing clinical trials studying anti-IL-1 strategies (except IL-1β strategies for lung cancer, shown in Table 2), alone or in combination, for cancer treatment

Therapy	Target	Tumor type	Recruitment status	Study type	**ClinicalTrials.gov** identifier	Trial name	Start date	Estimated completion date
Anakinra + chemotherapy	IL-1R	Pancreatic adeno-carcinoma	Active, not recruiting	Phase I	NCT02550327 [30]	–	January 16	August 2023
Anakinra + denosumab + everolimus	IL-1R, RANKL, and mTOR	Advanced, metastatic, recurrent or refractory cancer	Active, not recruiting	Phase I	NCT01624766 [31]	–	June 2012	June 2020
CAN04 + chemotherapy	IL-1RAP	NSCLC, pancreatic ductal adenocarcinoma, TNBC, CRC	Recruiting	Phase I/II	NCT03267316 [32]	CANFOUR	December 2017	June 2021
CAN04 + pembrolizumab	IL-1RAP, PD-1	NSCLC, urothelial carcinoma, malignant melanoma, HNSCC	Recruiting	Phase I	NCT04452214 [33]	–	September 2020	January 2022
Canakinumab	IL-1β	Chronic myelomonocytic leukemia or myelodysplastic syndrome	Recruiting	Phase II	NCT04239157 [34]	–	August 2020	December 2022
Canakinumab + spartalizumab	IL-1β, PD-1	Melanoma	Recruiting	Phase II	NCT03484923 [35]	PLATforM	September 2018	April 2022
Canakinumab + spartalizumab + LAG525	IL-1β, PD-1, LAG-3	TNBC	Recruiting	Phase Ib	NCT03742349 [36]	–	January 2019	January 2022
Canakinumab + spartalizumab + chemotherapy	IL-1β, PD-1	Pancreatic ductal adenocarcinoma	Recruiting	Phase Ib	NCT04581343 [37]	PanCAN-SR1	October 2020	March 2022
Canakinumab + spartalizumab	IL-1β, PD-1	RCC	Recruiting	Phase I	NCT04028245 [38]	SPARC-1	August 2019	December 2021
Gevokizumab + bevacizumab/ramucirumab/cabozantinib + chemotherapy	IL-1β, VEGF, VEGFR2	CRC, gastroesophageal cancer, RCC	Recruiting	Phase I	NCT03798626 [39]	–	May 2019	Jul 2024

Abbreviations: *CRC* colorectal cancer, *HNSCC* head and neck squamous cell carcinoma, *IL-1β* interleukin-1β, *IL-1R* interleukin-1 receptor, *IL-1RAP* interleukin-1 receptor accessory protein, *LAG-3* lymphocyte-activation gene 3, *NSCLC* non-small cell lung cancer, *PD-1* programmed death-1, *RANKL* receptor activator of nuclear factor kappa-Β ligand, *RCC* renal cell carcinoma, *TNBC* triple negative breast cancer, *VEGF* vascular endothelial growth factor, *VEGFR2* vascular endothelial growth factor receptor 2

## Interleukin-1β as an immunotherapeutic target

### The role of IL-1β in cancer

Two of the main mediators in the IL-1 family are the agonistic ligands IL-1α and IL-1β, with the antagonistic ligand IL-1 receptor antagonist (IL-1RA) binding to the same receptor, IL-1R [15]. Both IL-1α and IL-1β play pro-tumorigenic roles in several cancers. In vitro and in vivo data have shown that IL-1β in particular promotes migration and invasion by cancer cells, triggers a more aggressive cancer phenotype, drives immunosuppression, and induces local tumor development and angiogenesis [13, 15, 40, 41].

**Fig. 2:**
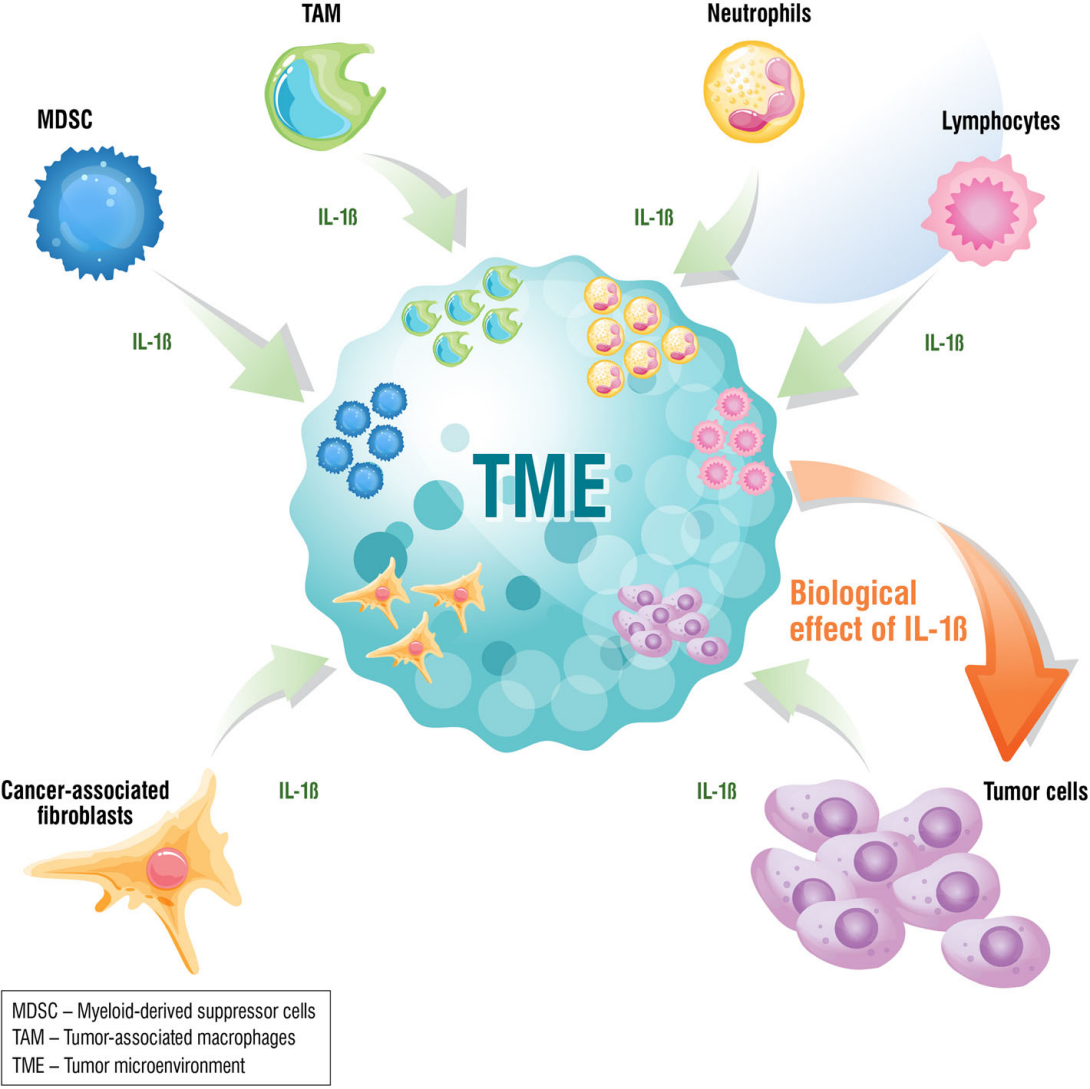
Role of IL-1β in the tumor microenvironment. Interleukin-1β (IL-1β) is a cytokine that plays diverse roles in the tumor microenvironment: it promotes tumor growth and invasiveness, induces angiogenesis, and creates an immunosuppressive environment. Tumor cell survival and proliferation may be promoted by high levels of circulating IL-1β due to chronic inflammation. Targeting IL-1β in the numerous pathways it is involved in within the tumor microenvironment results in an overall anti-tumor effect

IL-1 induces various changes in the components of the TME (Fig. 2). Cancer cells, in response to IL-1, produce factors that lead to angiogenesis and tumor progression [15]. Similarly, CAFs and adipocytes produce cancer-promoting factors and induce angiogenesis via IL-1β secretion [15]. IL-1β plays a role in determining the type of myeloid cell infiltrates present in the TME [42]. On this note, IL-1β is involved in the immunosuppression of the TME by activating and recruiting macrophages, inducing neutrophils to inhibit CD8^+^ T lymphocytes, suppressing NK effector function, and leading to production of angiogenic and pro-tumorigenic factors [15].

Suppression of IL-1β expression has been shown to reduce tumor growth and prevent shedding of tumor cells from the primary site into circulation [42, 43]. Blocking of IL-1β also increased the tumor infiltration by CD8^+^ T lymphocytes and decreased immunosuppression [42].

### Rationale for targeting IL-1β in lung cancer

Lung cancer is the second most commonly diagnosed form of cancer after breast cancer. Despite a dramatic decrease in lung cancer mortality in both men and women in the last few decades (since 1990 and 2002, respectively), it continues to be the leading cause of cancer death and its prognosis remains poor—only 19% of patients survive past 5 years [44].

Development of lung cancer is associated with a wide range of factors that lead to inflammation in the lungs, such as smoking, presence of COPD, pulmonary infections (e.g., tuberculosis), idiopathic pulmonary fibrosis, and occupational exposure to dust [45]. On this note, a markedly upregulated expression of inflammasome components, which led to IL-1β secretion, was found in human lung cancer tissue [46].

In vitro and in vivo studies have found IL-1β facilitates lung cancer metastasis by inducing cytokine production, angiogenesis, and tumor epithelial-to-mesenchymal transition, growth, invasion, and adhesion [43, 47–49]. The role of IL-1β in tumor progression is partially due to its effect on immune cells in the TME. A recent study found elevated IL-1β serum levels in lung cancer patients correlated with the high percentage of MDSCs and led to poor survival [50]. Macrophages isolated from human lung tumors have been found to be polarized toward the pro-tumoral M2 phenotype [51]. Elevated IL-1β produced by tumors from highly metastatic lung cancer cells induces macrophages to increase the expression of angiogenic and lymphangiogenic factors [52]. Additionally, secretion of IL-1β by lung cancer cells induces CD4^+^ T-cells to produce the tumor-promoting cytokine IL-22 [53]. Because of these effects, IL-1β also plays an important role in therapy resistance [54, 55].

In an in vivo study, injection of a carcinogen that promotes lung tumor development led to a higher population of inflammatory cells and higher serum levels of IL-1β and IL-6 [56]. Several inflammation markers present in the serum of patients have been associated with lung cancer risk [57]. In particular, a number of studies have identified IL-1β as a prognostic factor in lung cancer, with high levels of IL-1β in either serum or tumor tissue linked to poor survival [50, 58–60].

### Cantos trial: first evidence of anti-IL-1β therapy as a potential treatment for lung cancer

CANTOS was a multicenter, randomized, double-blind, placebo-controlled trial of 10,061 patients with a previous myocardial infarction, inflammatory atherosclerosis, and a persistent proinflammatory response—defined as a high-sensitivity CRP (hs-CRP) level over 2 mg/L. [61] Patients in the CANTOS trial were randomized to receive either placebo or one of 3 doses of canakinumab, a human monoclonal antibody that targets IL-1β and has anti-inflammatory effects. Canakinumab is currently FDA approved for the treatment of periodic fever syndromes and active systemic juvenile idiopathic arthritis [21].

Use of immunomodulatory agents has been linked to an increased risk of cancer development for transplant recipients or patients with inflammatory diseases [62, 63]. In this context, despite the focus of the CANTOS trial on atherosclerotic disease, a secondary analysis of these patients was conducted, revealing an unexpected finding: treatment with canakinumab resulted in a significantly lower incidence of lung cancer [64]. No significant reduction was found for the incidence of cancers at other sites. Moreover, the risk reduction for lung cancer was dose dependent (relative to canakinumab). Mortality for all cancers, and for lung cancer in particular, was lower in the combined canakinumab groups than in the placebo group.

In the CANTOS trial, canakinumab significantly reduced the serum levels of CRP (at 48 months) and IL-6 (only followed up for 12 months) in a dose-dependent manner [61]. CANTOS patients who were later diagnosed with lung cancer had significantly higher baseline CRP and IL-6 serum levels than patients who were not diagnosed with any cancer [64]. The baseline levels for these two biomarkers also trended with time to lung cancer diagnosis [65]. Levels of other inflammatory biomarkers were not significantly different between the lung cancer patient subset and the rest [65]. Taken together, these data suggest that targeting IL-1β plays a key role in reducing inflammation that could potentially promote the initiation and progression of lung cancer.

It is also important to note that while canakinumab was associated with a higher incidence of fatal infections in the CANTOS trial, patients were evaluated every 3 months in the trial while patients in lung cancer trials of canakinumab are evaluated every 3 weeks, allowing for prevention when identified early. It has also been shown that serious infections and infestations are dose dependent. In current trials of canakinumab in lung cancer, the 200-mg dose is used. Finally, despite the increased risk of fatal infection, there was no significant difference in all-cause mortality in the study [61]. In summary, considering the lower incidence of lung cancer and mortality, it is worthwhile to further investigate the value of canakinumab in lung cancer.

### Ongoing investigations with targeted IL-1β therapy in lung cancer

The unanticipated results from the CANTOS trial together with the preclinical findings on the role of IL-1β in lung cancer have led to the design of a number of clinical trials that are currently exploring IL-1β as a therapeutic target in lung cancer (Table 2). The CANOPY (CANakinumab Outcomes in Patients with NSCLC StudY) clinical program is studying the role of canakinumab alone or in combination with immunotherapy and/or chemotherapy across the majority of non-small cell lung cancer (NSCLC) settings.

**Table 2:** Ongoing clinical trials studying anti-IL-1β strategies, alone or in combination, for lung cancer treatment

Therapy	Target	Tumor type	Recruitment status	Study type	**ClinicalTrials.gov** identifier	Trial name	Start date	Estimated completion date
Canakinumab + pembrolizumab + chemotherapy	IL-1β + PD-1	NSCLC	Active, not recruiting	Phase III	NCT03631199 [66]	CANOPY-1	December 2018	September 2022
Canakinumab + chemotherapy	IL-1β	NSCLC	Active, not recruiting	Phase III	NCT03626545 [67]	CANOPY-2	January 2019	March 2022
Canakinumab +/− pembrolizumab	IL-1β +/− PD-1	NSCLC	Recruiting	Phase II	NCT03968419 [68]	CANOPY-N	November 2019	January 2022
Canakinumab	IL-1β	NSCLC	Recruiting	Phase III	NCT03447769 [69]	CANOPY-A	March 2018	January 2027
Canakinumab +/− PDR001	IL-1β +/− PD-1	NSCLC, TNBC, CRC	Active, not recruiting	Phase Ib	NCT02900664 [70]	–	August 2016	March 2021
Canakinumab + PDR001 + chemotherapy	IL-1β +/− PD-1	NSCLC	Active, not recruiting	Phase Ib	NCT03064854 [71]	ElevatION:NSCLC-101	May 2017	December 2021

Abbreviations: *CRC* colorectal cancer, *IL-1β* interleukin-1beta, *NSCLC* non-small cell lung cancer, *PD-1* programmed death-1, *PDR001* PD-1 inhibitor, aka spartalizumab, *TNBC* triple negative breast cancer

Half of CANOPY trials are studying the combination of anti-IL-1β with anti-PD-1 strategies. As mentioned previously, several anti-PD-1 therapies are approved for use in numerous cancer types, including lung cancer [12]. The combination of anti-IL-1β and anti-PD-1 therapy has shown in preclinical studies to have a synergistic effect that inhibited tumor growth and increased tumor infiltration by cytotoxic CD8^+^ lymphocytes [41, 42]. In two separate studies using pancreatic cancer or breast cancer mouse models, neutralization of IL-1β significantly enhanced the anti-tumor activity of anti-PD-1, and was accompanied by increased tumor infiltration of CD8^+^ T-cells [41, 42]. These studies demonstrate that blocking IL-1β reduces tumor progression through enhanced antitumor cell immunity. Furthermore, the synergistic action of IL-1β inhibition with anti-PD-1 improves tumor death, which may have significant clinical application [41, 42]. Unfortunately, such preclinical observations are not necessarily confirmed in clinical settings. For example, the CANOPY-2 study did not meet the primary endpoint of overall survival (OS); however, it confirmed the recommended phase III dose in the run-in phase and demonstrated canakinumab in combination with the chemotherapeutic drug, docetaxel, is safe. The study also suggested that identification of a predictive biomarker is likely needed to identify the right patient population, especially considering the heterogeneity because all participants had failed platinum-based chemotherapy and anti-PD-1/L1. The CANOPY-1 investigators have also announced that the study did not meet the primary endpoints of OS and progression-free survival (PFS); however, the study did demonstrate potentially clinically meaningful improvements in both the OS and PFS in prespecified subgroups of patients based on the baseline inflammatory biomarker (eg, hs-CRP), as well as other biomarker-defined subgroups. All these data support further evaluation of canakinumab in lung cancer.

## Conclusions

Immunotherapy is a rapidly growing methodology for use against a multitude of cancers. In the landscape of possible new therapies, IL-1β is a promising target. Recent data from the CANTOS trial highlighted a role for IL-1β in lung cancer development. Clinical outcomes of the CANTOS trial along with the ample in vitro and in vivo evidence of its role in tumor progression have led to the development of several clinical trials studying treatment of lung cancer based on anti-IL-1β therapy, either alone or in combination with anti-PD-1 therapy. While still pending definitive data, correlative studies and subgroup analyses from various clinical trials, we hope IL-1β inhibition opens a new avenue for lung cancer therapy through targeting pro-tumor inflammation.

## Data Availability

Not applicable.

## References

[1] Hanahan D, Weinberg RA (2011). Hallmarks of cancer: the next generation. Cell.

[2] Chen L, Deng H, Cui H, Fang J, Zuo Z, Deng J, Li Y, Wang X, Zhao L (2018). Inflammatory responses and inflammation-associated diseases in organs. Oncotarget.

[3] Bremnes RM, Al-Shibli K, Donnem T (2011). The role of tumor-infiltrating immune cells and chronic inflammation at the tumor site on cancer development, progression, and prognosis: emphasis on non-small cell lung cancer. J Thorac Oncol.

[4] Qian S, Golubnitschaja O, Zhan X (2019). Chronic inflammation: key player and biomarker-set to predict and prevent cancer development and progression based on individualized patient profiles. EPMA J.

[5] Del Prete A, Allavena P, Santoro G, Fumarulo R, Corsi MM, Mantovani A. Molecular pathways in cancer-related inflammation. Biochem Med. 2011:264–75. 10.11613/BM.2011.036. 10.11613/bm.2011.036 22420240

[6] Lu H, Ouyang W, Huang C (2006). Inflammation, a key event in cancer development. Mol Cancer Res.

[7] Baghban R, Roshangar L, Jahanban-Esfahlan R, Seidi K, Ebrahimi-Kalan A, Jaymand M, Kolahian S, Javaheri T, Zare P (2020). Tumor microenvironment complexity and therapeutic implications at a glance. Cell Commun Signal.

[8] Hinshaw DC, Shevde LA (2019). The tumor microenvironment innately modulates cancer progression. Cancer Res.

[9] Cui Y, Guo G (2016). Immunomodulatory function of the tumor suppressor p53 in host immune response and the tumor microenvironment. Int J Mol Sci.

[10] Zhang J, Shi Z, Xu X, Yu Z, Mi J (2019). The influence of microenvironment on tumor immunotherapy. FEBS J.

[11] Greten FR, Grivennikov SI. Inflammation and cancer: triggers, mechanisms, and consequences. Immunity. 2019;51(1):27–41. 10.1016/j.immuni.2019.06.025. 10.1016/j.immuni.2019.06.025 PMC6831096 31315034

[12] Christofi T, Baritaki S, Falzone L, Libra M, Zaravinos A (2019). Current perspectives in cancer immunotherapy. Cancers (Basel).

[13] Gelfo V, Romaniello D, Mazzeschi M, Sgarzi M, Grilli G, Morselli A, Manzan B, Rihawi K, Lauriola M (2020). Roles of il-1 in cancer: from tumor progression to resistance to targeted therapies. Int J Mol Sci.

[14] Mantovani A, Dinarello CA, Molgora M, Garlanda C (2019). Interleukin-1 and related cytokines in the regulation of inflammation and immunity. Immunity..

[15] Zhang W, Borcherding N, Kolb R (2020). IL-1 signaling in tumor microenvironment. Adv Exp Med Biol.

[16] Fridman WH, Zitvogel L, Sautès-Fridman C, Kroemer G (2017). The immune contexture in cancer prognosis and treatment. Clin Oncol.

[17] Becht E, Giraldo NA, Lacroix L, et al. Estimating the population abundance of tissue-infiltrating immune and stromal cell populations using gene expression. Genome Biol. 2016;17(1):218. 10.1186/s13059-016-1070-5. 10.1186/s13059-016-1113-y PMC5134277 27908289

[18] Jiang Y, Chen M, Nie H, Yuan Y. PD-1 and PD-L1 in cancer immunotherapy: clinical implications and future considerations. Hum Vaccines Immunother. 2019;15(5):1111–22. 10.1080/21645515.2019.1571892. 10.1080/21645515.2019.1571892 PMC6605868 30888929

[19] Pardoll DM (2012). The blockade of immune checkpoints in cancer immunotherapy. Nat Rev Cancer.

[20] Proleukin® (aldesleukin). San Diego, CA: Prometheus Laboratories, Inc.; 2012. https://www.accessdata.fda.gov/drugsatfda_docs/label/2012/103293s5130lbl.pdf. Accessed 9 Aug 2021.

[21] Ilaris® (canakinumab) [package insert]. East Hanover, NJ: Novartis Pharmaceuticals Corporation; 2016. https://www.accessdata.fda.gov/drugsatfda_docs/label/2016/BLA125319_858687lbl.pdf. Accessed 9 Aug 2021.

[22] Kineret® (anakinra). Stockholm, Sweden: Swedish Orphan Biovitrum AB; 2012. https://www.accessdata.fda.gov/drugsatfda_docs/label/2012/103950s5136lbl.pdf. Accessed 9 Aug 2021.

[23] Arcalyst™ (rilonacept). Tarrytown, NY: Regeneron Pharmaceuticals Inc; 2008. https://www.accessdata.fda.gov/drugsatfda_docs/label/2008/125249lbl.pdf. Accessed 9 Aug 2021.

[24] Wu TC, Xu K, Martinek J, Young RR, Banchereau R, George J, Turner J, Kim KI, Zurawski S, Wang X, Blankenship D, Brookes HM, Marches F, Obermoser G, Lavecchio E, Levin MK, Bae S, Chung CH, Smith JL, Cepika AM, Oxley KL, Snipes GJ, Banchereau J, Pascual V, O'Shaughnessy J, Palucka AK (2018). IL1 receptor antagonist controls transcriptional signature of inflammation in patients with metastatic breast cancer. Cancer Res.

[25] Isambert N, Hervieu A, Rébé C (2018). Fluorouracil and bevacizumab plus anakinra for patients with metastatic colorectal cancer refractory to standard therapies (IRAFU): a single-arm phase 2 study. Oncoimmunology.

[26] Lust JA, Lacy MQ, Zeldenrust SR, Dispenzieri A, Gertz MA, Witzig TE, Kumar S, Hayman SR, Russell SJ, Buadi FK, Geyer SM, Campbell ME, Kyle RA, Rajkumar SV, Greipp PR, Kline MP, Xiong Y, Moon-Tasson LL, Donovan KA (2009). Induction of a chronic disease state in patients with smoldering or indolent multiple myeloma by targeting interleukin 1β-induced interleukin 6 production and the myeloma proliferative component. Mayo Clin Proc.

[27] Lust JA, Lacy MQ, Zeldenrust SR, Witzig TE, Moon-Tasson LL, Dinarello CA, Donovan KA (2016). Reduction in C-reactive protein indicates successful targeting of the IL-1/IL-6 axis resulting in improved survival in early stage multiple myeloma. Am J Hematol.

[28] Wirth LJ, Eigendorff E, Capdevila J (2018). Phase I/II study of spartalizumab (PDR001), an anti-PD1 mAb, in patients with anaplastic thyroid cancer. J Clin Oncol.

[29] Naing A, Gainor JF, Gelderblom H, et al. A first-in-human phase 1 dose escalation study of spartalizumab (PDR001), an anti-PD-1 antibody, in patients with advanced solid tumors. J Immunother Cancer. 2020;8(1). 10.1136/jitc-2020-000530. 10.1136/jitc-2020-000530 PMC7073791 32179633

[30] Gemcitabine, Nab-Paclitaxel, Cisplatin and Anakinra Treatment on Patients With Pancreatic Cancer. ClinicalTrials.gov Identifier: https://clinicaltrials.gov/show/NCT02550327. Accessed 9 Aug 2021.

[31] Everolimus and Anakinra or Denosumab in Treating Participants With Relapsed or Refractory Advanced Cancers. ClinicalTrials.gov Identifier: NCT01624766. https://clinicaltrials.gov/ct2/show/NCT01624766. Accessed 9 Aug 2021.

[32] A First-in-Human Study of CAN04 in Patients With Solid Malignant Tumors. Clinicaltrials.gov. Identifier: NCT03267316. https://clinicaltrials.gov/show/NCT03267316

[33] A Study of the Safety and Tolerance of CAN04 in Combination With Pembrolizumab in Subjects With Solid Tumors. ClinicalTrials.gov Identifier: NCT04452214. https://clinicaltrials.gov/ct2/show/NCT04452214. Accessed 9 Aug 2021.

[34] Canakinumab and Anacitidine for the Treatment of Low or Intermediate Risk Myelodysplastic Syndrome and Chronic Myelomonocytic Leukemia. ClinicalTrials.gov Identifier: NCT04239157. https://clinicaltrials.gov/ct2/show/NCT04239157. Accessed 9 Aug 2021.

[35] Study of Efficacy and Safety of Novel Spartalizumab Combinations in Patients With Previously Treated Unresectable or Metastatic Melanoma (PLATforM). ClinicalTrials.gov Identifier: NCT03484923. https://clinicaltrials.gov/ct2/show/NCT03484923. Accessed 9 Aug 2021.

[36] Study of Safety and Efficacy of Novel Immunotherapy Combinations in Patients With Triple Negative Breast Cancer (TNBC). ClinicalTrials.gov Identifier: NCT03742349. https://clinicaltrials.gov/ct2/show/NCT03742349. Accessed 9 Aug 2021.

[37] A Phase 1B Study of Canakinumab, Spartalizumab, Nab-paclitaxel, and Gemcitabine in Metastatic PC Patients (PanCAN-SR1). ClinicalTrials.gov Identifier: NCT04581343. https://clinicaltrials.gov/ct2/show/ NCT04581343. Accessed 9 Aug 2021.

[38] A Study of Combination Spartalizumab and Canakinumab in Patients With Localized Clear Cell Renal Cell Carcinoma (SPARC-1). ClinicalTrials.gov Identifier: NCT04028245. https://clinicaltrials.gov/ct2/show/NCT04028245. Accessed 9 Aug 2021.

[39] Gevokizumab With Standard of Care Anti-cancer Therapies for Metastatic Colorectal, Gastroesophageal, and Renal Cancers. ClinicalTrials.gov Identifier: NCT03798626. https://clinicaltrials.gov/ct2/show/NCT03798626. Accessed 9 Aug 2021.

[40] Voronov E, Shouval DS, Krelin Y, Cagnano E, Benharroch D, Iwakura Y, Dinarello CA, Apte RN (2003). IL-1 is required for tumor invasiveness and angiogenesis. Proc Natl Acad Sci U S A.

[41] Das S, Shapiro B, Vucic EA, Vogt S, Bar-Sagi D (2020). Tumor cell-derived IL1β promotes desmoplasia and immune suppression in pancreatic cancer. Cancer Res.

[42] Kaplanov I, Carmi Y, Kornetsky R, Shemesh A, Shurin GV, Shurin MR, Dinarello CA, Voronov E, Apte RN (2019). Blocking IL-1β reverses the immunosuppression in mouse breast cancer and synergizes with anti–PD-1 for tumor abrogation. Proc Natl Acad Sci U S A.

[43] Tulotta C, Lefley DV, Freeman K, Gregory WM, Hanby AM, Heath PR, Nutter F, Wilkinson JM, Spicer-Hadlington AR, Liu X, Bradbury SMJ, Hambley L, Cookson V, Allocca G, Kruithof de Julio M, Coleman RE, Brown JE, Holen I, Ottewell PD (2019). Endogenous production of IL1B by breast cancer cells drives metastasis and colonization of the bone microenvironment. Clin Cancer Res.

[44] Siegel RL, Miller KD, Jemal A (2020). Cancer statistics, 2020. CA Cancer J Clin.

[45] O’Callaghan DS, O’Donnell D, O’Connell F, O’Byrne KJ (2010). The role of inflammation in the pathogenesis of non-small cell lung cancer. J Thorac Oncol.

[46] Kong H, Wang Y, Zeng X, Wang Z, Wang H, Xie W (2015). Differential expression of inflammasomes in lung cancer cell lines and tissues. Tumor Biol.

[47] Yano S, Nokihara H, Yamamoto A, Goto H, Ogawa H, Kanematsu T, Miki T, Uehara H, Saijo Y, Nukiwa T, Sone S (2003). Multifunctional interleukin-1β promotes metastasis of human lung cancer cells in SCID mice via enhanced expression of adhesion-, invasion- and angiogenesis-related molecules. Cancer Sci.

[48] Saijo Y, Tanaka M, Miki M, Usui K, Suzuki T, Maemondo M, et al. Proinflammatory cytokine IL-1β promotes tumor growth of Lewis lung carcinoma by induction of angiogenic factors: in vivo analysis of tumor-stromal interaction. J Immunol. 2002;169(1):469–75. 10.4049/jimmunol.169.1.469. 10.4049/jimmunol.169.1.469 12077278

[49] Li R, Ong SL, Tran LM (2020). Chronic IL-1β-induced inflammation regulates epithelial-to-mesenchymal transition memory phenotypes via epigenetic modifications in non-small cell lung cancer. Sci Rep.

[50] Barrera L, Montes-Servín E, Hernandez-Martinez JM, Orozco-Morales M, Montes-Servín E, Michel-Tello D, Morales-Flores RA, Flores-Estrada D, Arrieta O (2018). Levels of peripheral blood polymorphonuclear myeloid-derived suppressor cells and selected cytokines are potentially prognostic of disease progression for patients with non-small cell lung cancer. Cancer Immunol Immunother.

[51] Lambrechts D, Wauters E, Boeckx B, Aibar S, Nittner D, Burton O, Bassez A, Decaluwé H, Pircher A, van den Eynde K, Weynand B, Verbeken E, de Leyn P, Liston A, Vansteenkiste J, Carmeliet P, Aerts S, Thienpont B (2018). Phenotype molding of stromal cells in the lung tumor microenvironment. Nat Med.

[52] Watari K, Shibata T, Kawahara A, Sata KI, Nabeshima H, Shinoda A, Abe H, Azuma K, Murakami Y, Izumi H, Takahashi T, Kage M, Kuwano M, Ono M (2014). Tumor-derived interleukin-1 promotes lymphangiogenesis and lymph node metastasis through M2-type macrophages. PLoS One.

[53] Voigt C, May P, Gottschlich A, Markota A, Wenk D, Gerlach I, Voigt S, Stathopoulos GT, Arendt KAM, Heise C, Rataj F, Janssen KP, Königshoff M, Winter H, Himsl I, Thasler WE, Schnurr M, Rothenfußer S, Endres S, Kobold S (2017). Cancer cells induce interleukin-22 production from memory CD4+ T cells via interleukin-1 to promote tumor growth. Proc Natl Acad Sci U S A.

[54] Huang J, Lan X, Wang T, Lu H, Cao M, Yan S, Cui Y, Jia D, Cai L, Xing Y (2020). Targeting the IL-1β/EHD1/TUBB3 axis overcomes resistance to EGFR-TKI in NSCLC. Oncogene..

[55] Gelfo V, Mazzeschi M, Grilli G (2018). A novel role for the interleukin-1 receptor axis in resistance to anti-EGFR therapy. Cancers (Basel).

[56] Narayan C, Kumar A (2012). Constitutive over expression of IL-1β, IL-6, NF-κB, and Stat3 is a potential cause of lung tumorgenesis in urethane (ethyl carbamate) induced Balb/c mice. J Carcinog.

[57] Shiels MS, Pfeiffer RM, Hildesheim A, Engels EA, Kemp TJ, Park JH, Katki HA, Koshiol J, Shelton G, Caporaso NE, Pinto LA, Chaturvedi AK (2013). Circulating inflammation markers and prospective risk for lung cancer. J Natl Cancer Inst.

[58] McLoed AG, Sherrill TP, Cheng DS (2016). Neutrophil-derived IL-1β impairs the efficacy of NF-κB inhibitors against lung cancer. Cell Rep.

[59] Kim JW, Koh Y, Kim DW, Ahn YO, Kim TM, Han SW, Oh DY, Lee SH, Im SA, Kim TY, Heo DS, Bang YJ (2013). Clinical implications of VEGF, TGF-β1, and IL-1β in patients with advanced non-small cell lung cancer. Cancer Res Treat.

[60] Millares L, Barreiro E, Cortes R, Martinez-Romero A, Balcells C, Cascante M, Enguita AB, Alvarez C, Rami-Porta R, Sánchez de Cos J, Seijo L, Monsó E, Grupo Colaborativo en Cáncer de Pulmón CIBERES- CIBERONC- SEPAR - Plataforma Biobanco Pulmonar (2018). Tumor-associated metabolic and inflammatory responses in early stage non-small cell lung cancer: local patterns and prognostic significance. Lung Cancer.

[61] Ridker PM, Everett BM, Thuren T, MacFadyen JG, Chang WH, Ballantyne C, Fonseca F, Nicolau J, Koenig W, Anker SD, Kastelein JJP, Cornel JH, Pais P, Pella D, Genest J, Cifkova R, Lorenzatti A, Forster T, Kobalava Z, Vida-Simiti L, Flather M, Shimokawa H, Ogawa H, Dellborg M, Rossi PRF, Troquay RPT, Libby P, Glynn RJ (2017). Antiinflammatory therapy with canakinumab for atherosclerotic disease. N Engl J Med.

[62] Beyaert R, Beaugerie L, Van Assche G (2013). Cancer risk in immune-mediated inflammatory diseases (IMID). Mol Cancer.

[63] Bojinca V, Janta I (2012). Rheumatic diseases and malignancies. Maedica (Buchar).

[64] Ridker PM, MacFadyen JG, Thuren T (2017). Effect of interleukin-1β inhibition with canakinumab on incident lung cancer in patients with atherosclerosis: exploratory results from a randomised, double-blind, placebo-controlled trial. Lancet.

[65] Wong CC, Baum J, Silvestro A (2020). Inhibition of IL-1β by canakinumab may be effective against diverse molecular subtypes of lung cancer: an exploratory analysis of the CANTOS trial. Cancer Res.

[66] Study of Efficacy and Safety of Pembrolizumab Plus Platinum-based Doublet Chemotherapy With or Without Canakinumab in Previously Untreated Locally Advanced or Metastatic Non-squamous and Squamous NSCLC Subjects (CANOPY-1). ClinicalTrials.gov Identifier: NCT03631199. https://clinicaltrials.gov/ct2/show/NCT03631199. Accessed 9 Aug 2021.

[67] Phase III Study Evaluating Efficacy and Safety of Canakinumab in Combination With Docetaxel in Adult Subjects With Non-small Cell Lung Cancers as a Second or Third Line Therapy (CANOPY-2). ClinicalTrials.gov Identifier: NCT03626545. https://clinicaltrials.gov/ct2/show/NCT03626545. Accessed 9 Aug 2021.

[68] This Study Will Evaluate the Effect of Canakinumab or Pembrolizumab Given as Monotherapy or in Combination as Neo-adjuvant Treatment for Subjects With Early Stages NSCLC. (CANOPY-N). ClinicalTrials.gov Identifier: NCT03968419. https://clinicaltrials.gov/ct2/show/NCT03968419. Accessed 9 Aug 2021.

[69] Study of Efficacy and Safety of Canakinumab as Adjuvant Therapy in Adult Subjects With Stages AJCC/UICC v. 8 II-IIIA and IIIB (T>5cm N2) Completely Resected Non-small Cell Lung Cancer Acronym: CANOPY-A (Canopy-A). ClinicalTrials.gov Identifier: NCT03447769. https://clinicaltrials.gov/ct2/show/NCT03447769. Accessed 9 Aug 2021.

[70] A Study of PDR001 in Combination With CJM112, EGF816, Ilaris® (Canakinumab) or Mekinist® (Trametinib). ClinicalTrials.gov Identifier: NCT02900664. https://clinicaltrials.gov/ct2/show/NCT02900664. Accessed 9 Aug 2021.

[71] PDR001 in Combination With Platinum-doublet Chemotherapy and Other Immunology Agents in PD-L1 Unselected, Metastatic NSCLC Patients. ClinicalTrials.gov Identifier: NCT03064854. https://clinicaltrials.gov/ct2/show/NCT03064854. Accessed 9 Aug 2021.

